# Esophageal cancer in an adult with congenital esophageal stenosis: a case report

**DOI:** 10.1186/s40792-024-01858-1

**Published:** 2024-03-12

**Authors:** Yushi Fujiwara, Hidehiko Kitagami, Tomohiro Kikkawa, Keita Sakashita, Takaya Kusumi, Yasunori Nishida

**Affiliations:** Department of Gastroenterological Surgery, Keiyukai Sapporo Hospital, Minami1-1, Hondori 9-Chome, Shiroishi-Ku, Sapporo, 003-0026 Japan

**Keywords:** Congenital esophageal stenosis, Esophageal cancer, Fibromuscular thickening, Adult

## Abstract

**Background:**

Congenital esophageal stenosis (CES) is a rare condition. We encountered a case of esophageal cancer that developed in an adult with persistent CES. Although many studies have investigated the therapeutic outcomes and performed surveillance for symptoms after treatment for CES, few have performed long-term surveillance or reported on the development of esophageal cancer. We report this case because it is extremely rare and has important implications.

**Case presentation:**

A 45-year-old woman with worsening dysphagia was transferred to our hospital. The patient was diagnosed with CES at 5 years of age and underwent surgery at another hospital. The patient underwent esophageal dilatation for stenosis at 36 years of age. Esophagoscopy performed at our hospital revealed a circumferential ulcerated lesion and stenosis 15–29 cm from the incisors. Histological examination of the biopsy specimen revealed squamous cell carcinoma. Computed tomography (CT) revealed abnormal circumferential wall thickening in parts of the cervical and almost the entire thoracic esophagus. ^18^F-fluorodeoxyglucose-positron emission tomography-CT revealed increased uptake in the cervical and upper esophagus. No uptake was observed in the muscular layers of the middle or lower esophagus. Based on these findings, the patient was diagnosed with clinical stage IVB cervical and upper esophageal cancer (T3N1M1 [supraclavicular lymph nodes]). The patient underwent a total esophagectomy after neoadjuvant chemotherapy. The esophagus was markedly thickened and tightly adhered to the adjacent organs. Severe fibrosis was observed around the trachea. Marked thickening of the muscular layer was observed throughout the esophagus; histopathological examination revealed that this thickening was due to increased smooth muscle mass. No cartilage, bronchial epithelium, or glands were observed. The carcinoma extended from the cervical to the middle esophagus, oral to the stenotic region. Finally, we diagnosed the patient with esophageal cancer developing on CES of the fibromuscular thickening type.

**Conclusions:**

Chronic mechanical and chemical irritations are believed to cause cancer of the upper esophagus oral to a persistent CES, suggesting the need for long-term surveillance that focuses on residual stenosis and cancer development in patients with CES.

## Background

Congenital esophageal stenosis (CES) is a rare malformation [1–6]. Almost all patients with CES are diagnosed and treated for esophageal stenosis during childhood [6, 7], and they can achieve remission [1]. However, some patients develop protracted stenosis, even after treatment [2].

Here, we report our encounter of a case of esophageal cancer that developed in an adult with persistent CES. Although therapeutic outcomes and symptomatic surveillance after treatment for CES have been investigated in many studies [1–3, 8], few have reported on long-term surveillance and the development of esophageal cancer. We report this case because it is extremely rare and has important implications.

## Case presentation

**Fig. 1:**
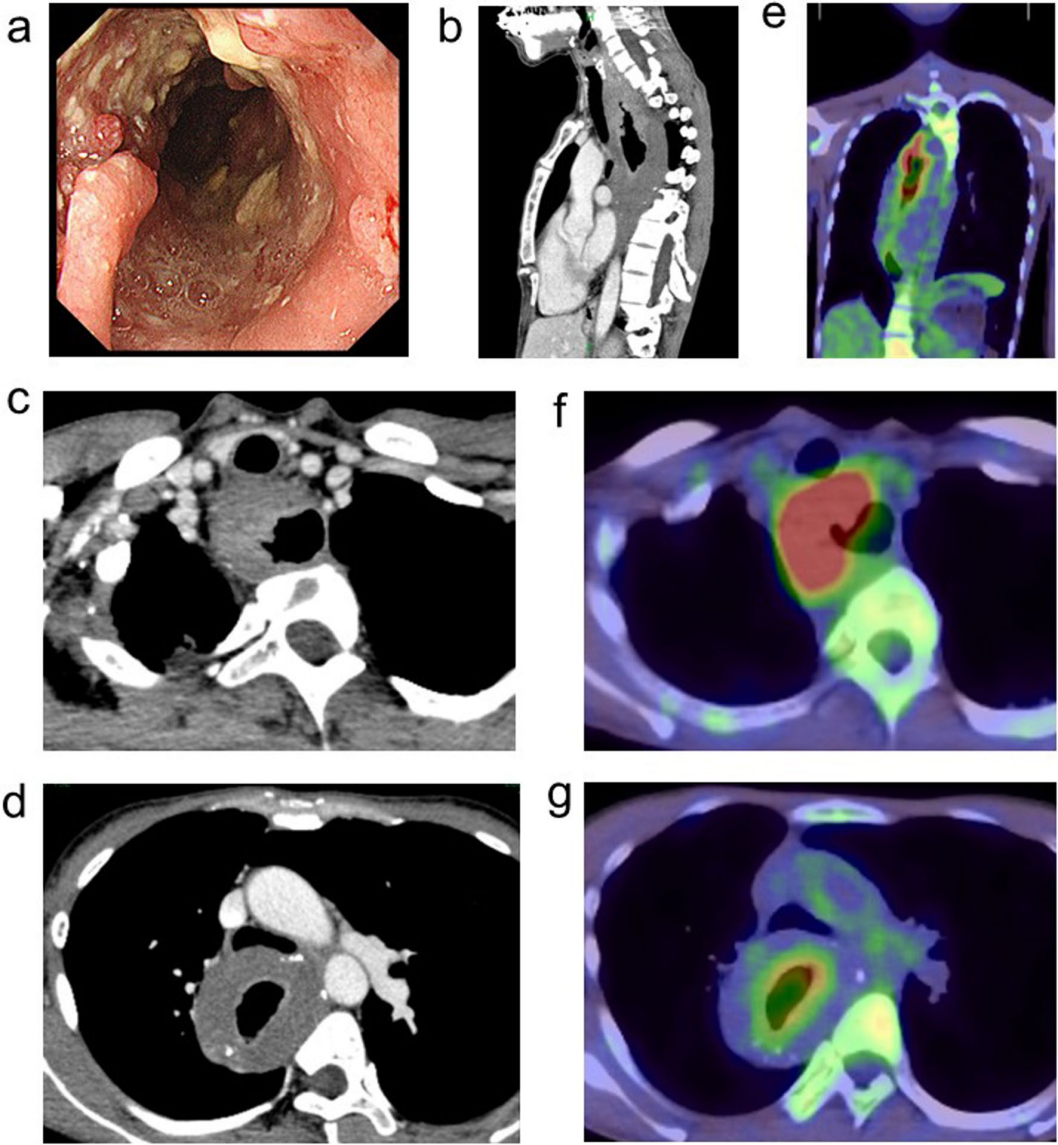
Findings before treatment. **a** Esophagoscopy shows a circumferential ulcerated lesion and stenosis 15–29 cm from the incisors. **b**–**d** Contrast-enhanced computed tomography (CT) image: abnormal circumferential wall thickening in parts of the cervical and most of the thoracic esophagus. Cervical lesion invading the adventitia (cT3). ** e–g** Positron emission tomography (PET)-CT images show uptake in the cervical and upper esophagus and no uptake in the muscular layer of the middle and lower esophagus

A 45-year-old woman was transferred to our hospital to receive treatment for cervical esophageal cancer. The patient had worsening dysphagia for 2 months and could not eat solid or liquid food at admission; she also had a history of CES. The patient was diagnosed with CES at 5 years of age and underwent surgery at another hospital. Details of the surgical procedure were not available. The patient had undergone esophageal dilatation for esophageal stenosis at 36 years of age. The patient had never smoked tobacco and drank alcohol socially without experiencing alcohol flushing. Esophagoscopy performed at our hospital revealed a circumferential ulcerated lesion and stenosis 15–29 cm from the incisors (Fig. 1a).

Histological examination of the biopsy specimen from the cervical esophagus revealed squamous cell carcinoma. A barium swallow was not performed owing to the risk of aspiration. Contrast-enhanced computed tomography (CT) revealed abnormal circumferential wall thickening in parts of the cervical and most of the thoracic esophagus. Among these, the cervical lesions invaded the adventitia (cT3) (Fig. 1b–d). Metastases to the bilateral recurrent laryngeal nerves and left supraclavicular lymph nodes (LNs) were observed (cN1M1). ^18^F-fluorodeoxyglucose-positron emission tomography (PET)-CT showed uptake in the cervical and upper esophagus, whereas no uptake was observed in the muscular layers of the middle and lower esophagus (Fig. 1e–g). The marked wall thickening observed in parts of the middle and lower esophagus was believed to result from hypertrophy due to the CES. Based on these findings, the patient was diagnosed with clinical stage IVB cervical and upper esophageal cancer (T3N1M1), according to the American Joint Committee on Cancer/Tumor-Node-Metastasis Staging System, eighth edition [9].

The patient opted to undergo neoadjuvant chemotherapy followed by surgery and received docetaxel, cisplatin, and 5-fluorouracil (DCF) therapy. After three cycles of DCF, the size of both the primary lesion and metastatic LNs was reduced.

The patient underwent the surgery as planned. A total esophagectomy was performed via open thoracotomy because the esophageal tumor was large. The esophagus was markedly thickened and tightly adhered to adjacent organs. Because the fibrosis was severe, especially around the trachea, we could not recognize the anatomy around the trachea. Hence, we could not perform radical lymph node dissection in the upper mediastinum. Free jejunal transfer with microvascular anastomosis was required for reconstruction because the gastric tube did not reach the anastomotic level.

After esophagectomy, necrosis of the transferred free jejunum occurred as a complication, and reoperation was required. Owing to these invasive surgeries, the patient developed methicillin-resistant *Staphylococcus aureus* pneumonia and unfortunately died of multiple organ failure on postoperative day 67.

**Fig. 2:**
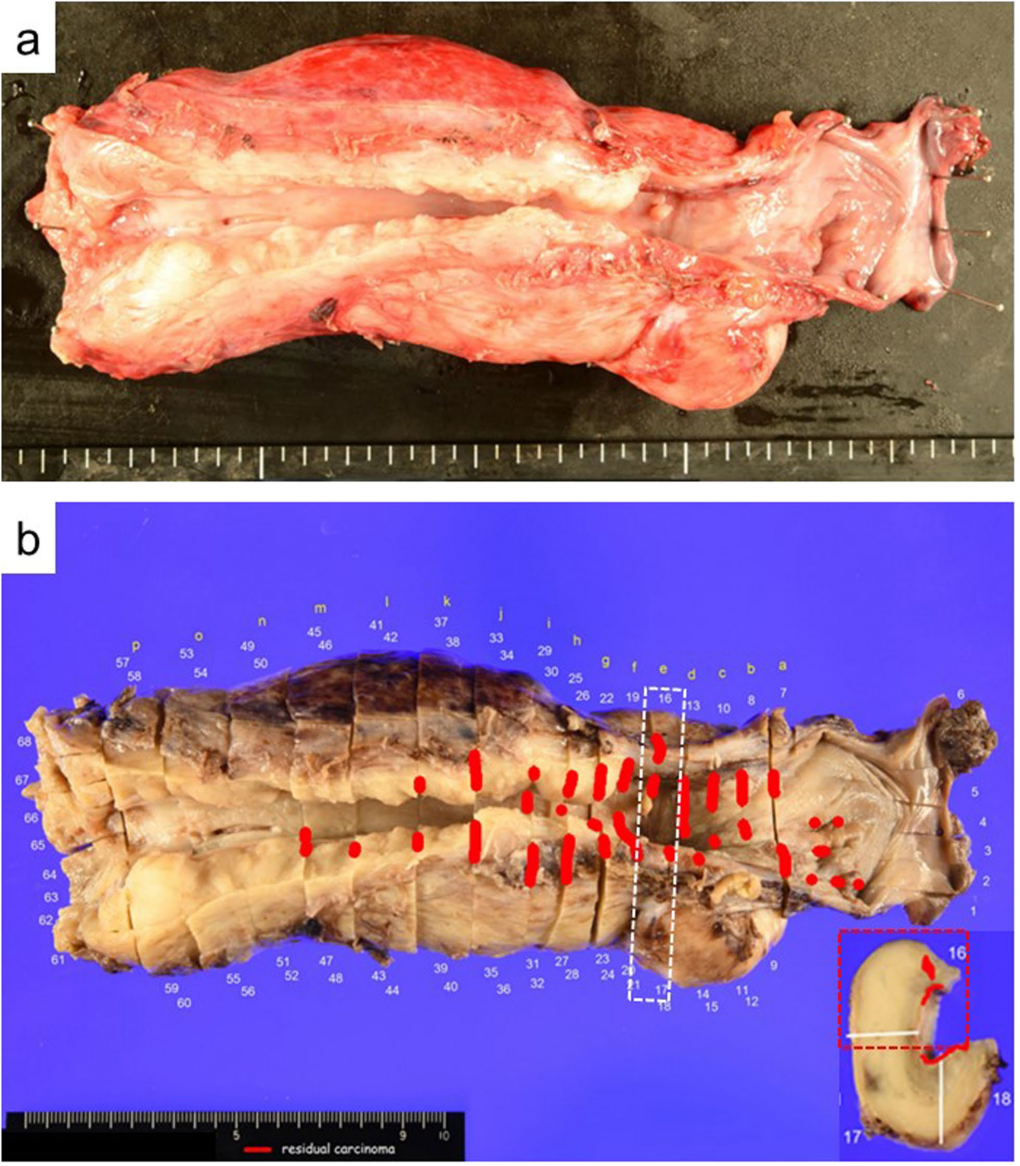
Macroscopic findings of the resected esophagus. **a** The marked thickness of the muscular layer of the entire esophagus is observed. **b** Squamous cell carcinoma extending from the cervical to the middle esophagus (red line)

**Fig. 3:**
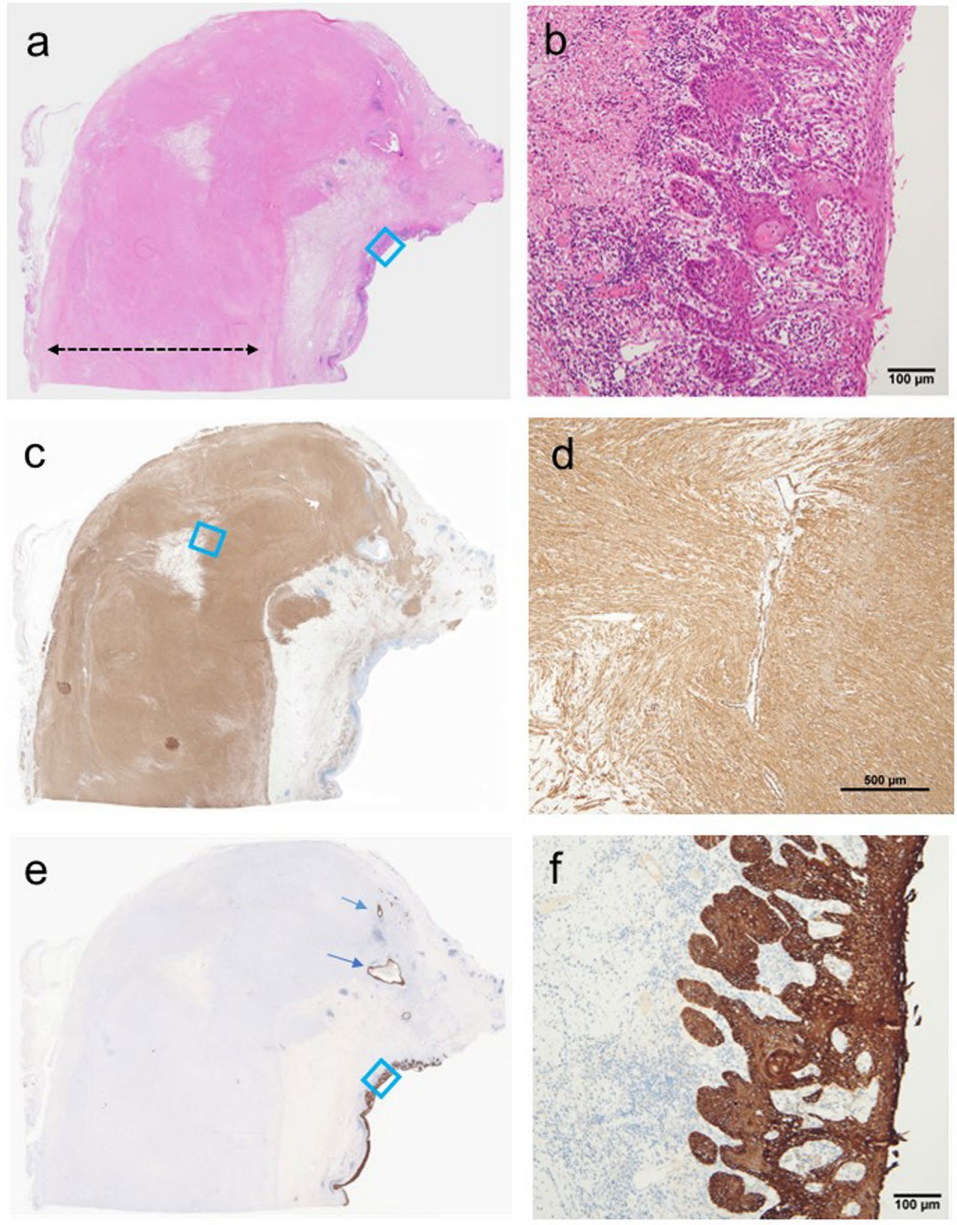
Histopathological findings (area enclosed by the dashed red line in Fig. 2b). **a**, **b** Hematoxylin and eosin (H&E) staining findings. The wall thickening is attributable to the thickness of the muscular layer (area enclosed by the dashed black line). The esophageal tumor is a squamous cell carcinoma. **c**, **d** α-smooth muscle actin [α-SMA] staining findings. The increased thickness of the muscular layer is caused by the increased number of smooth muscle. **e**, **f** Cytokeratin 34βE12 staining findings. Squamous cell carcinoma invading the muscularis propria (blue arrow)

Macroscopic examination of the resected esophagus revealed marked wall thickening in the entire esophagus (Fig. 2a and b). This wall thickening was due to the thickening of the muscular layer (Fig. 3a). The smooth muscle mass of the muscular layer was increased (Fig. 3c and d). No cartilage, bronchial epithelium, or glands were observed in the resected specimens. Based on these findings, the patient was considered to have the fibromuscular thickening type of CES [1–4, 9]. The squamous cell carcinoma affected the cervical to the middle esophagus (Fig. 2b) and invaded the muscularis propria (ypT2) (Figs. 3b, e, and f). No metastatic LNs were detected (ypN0). Finally, we diagnosed this patient with esophageal cancer developing on CES of the fibromuscular thickening type.

## Discussion

CES is a rare condition with an incidence rate of 1/25,000–50,000 live births [1–6]. Almost all patients with CES are diagnosed and treated for esophageal stenosis during childhood [6, 7]. The therapeutic options include endoscopic surgery, dilatation, and transthoracic surgery [1–3]. Some patients with mild symptoms are diagnosed with CES during adulthood [5–7]; however, proving whether adults with esophageal stenosis truly have CES is difficult. Therefore, excluding postnatally acquired stenoses (peptic, caustic, infectious, and neoplastic), extrinsic compression, and achalasia is essential [3]. In this case, the patient was diagnosed with CES at 5 years of age and underwent surgery, although the details were not provided. Subsequently, the patient underwent esophageal dilatation for esophageal stenosis at 36 years of age, suggesting that esophageal stenosis persists until adulthood. Notably, more than half of patients who undergo surgery require dilatation after surgery for post-surgical anastomotic stenosis or residual stenosis [1].

CES is histologically categorized into three types: (a) ectopic tracheobronchial remnants (TBR), (b) membranous web (MW), and (c) fibromuscular thickening or fibromuscular stenosis (FMS) [1–4, 9]. The patient in the present case was diagnosed with FMS based on the clinical and histopathological findings. Fibromuscular thickening stenosis mainly affects the middle or lower thirds of the esophagus [3]. In our case, fibromuscular thickening was observed on histopathological examination of the long segment between the upper and lower esophagus.

To the best of our knowledge, only Tabira et al. [5] have reported a case of esophageal cancer arising on CES (Table 1). In their case, cancer lesions occurred on the stricture, and chronic mechanical stimulation by food was speculated to be a carcinogenic factor. We believe that food may have mechanically stimulated the oral side of the stenotic segment for a prolonged period. Considering the long duration of symptoms and long, narrow area, the impact of the mechanical stimulation is believed to be significant. Several mechanisms for the development of squamous cell carcinoma of the esophagus in patients with esophageal achalasia, which is similarly associated with stasis within the esophagus, have been proposed [10, 11]. Continuous chemical irritation due to salivary stasis and food decomposition in the esophagus plays a major role in inducing inflammation. Additionally, nitrosamine production from bacterial overgrowth in the esophageal lumen due to food stasis leads to inflammation. Under these conditions, chronic inflammation is believed to contribute to the development of cancer. Similarly, in our case, we believe that chronic mechanical and chemical irritation may have caused the cancer to develop in the upper esophagus and oral side of the stenotic segment.

**Table 1:** Esophageal cancer in an adult with CES

Year	Author	Age	Sex	Type of CES	Duration from diagnosis of CES	Location of CES	Location of the cancer	Treatment	TNM
2002	Tabira et al.	65	Male	FMS	6 years	Upper esophagus	On the stricture	Esophagectomy	T1N0M0
	Our case	45	Female	FMS	40 years	Entire esophagus	From the cervical to the middle esophagus	Esophagectomy	T3N1M1

*CES* congenital esophageal stenosis, *FMS* fibromuscular thickening or fibromuscular stenosis

To date, some studies have reported the mid- and long-term outcomes of CES treatment [1, 2, 8]. However, long-term surveillance until adulthood has not been performed. Particularly, cancer developing in patients diagnosed with CES during childhood has not been reported. Endoscopic dilatation, or surgery, is the initial treatment for stenosis due to CES. Surgery tends to be performed in patients for whom dilatation is ineffective. However, in some patients, symptoms of stenosis persist even after these treatments [1, 2]. In these cases, long-term stenosis persists from childhood, and there is prolonged mechanical and chemical irritation of the esophagus. In patients with persistent stenosis, the risk of cancer may increase. Early esophageal cancer can be treated using less invasive treatments such as endoscopic resection or chemoradiation therapy, implying the importance of early detection of cancer. Therefore, it is important to determine whether stenosis persists in patients with CES. Long-term surveillance for cancer is required if the stenosis persists.

## Conclusion

We encountered a case of esophageal cancer that developed in an adult with CES of the FMS type. Long-term surveillance that focuses on residual stenosis and cancer development should be conducted in patients with CES. Additionally, data accumulation during long-term follow-up of patients with CES is required.

## Data Availability

Not applicable.

## Abbreviations

CES: Congenital esophageal stenosis
CT: Computed tomography
DCF: Docetaxel, cisplatin, and 5-fluorouracil
FMS: Fibromuscular thickening or fibromuscular stenosis
LNs: Lymph nodes
MW: Membranous web
PET: ^18^F-fluorodeoxyglucose-positron emission tomography
TBR: Ectopic tracheobronchial remnants
